# Effect of Oral Administration of *Lactiplantibacillus plantarum* SNK12 on Temporary Stress in Adults: A Randomized, Placebo-Controlled, Double-Blind, Parallel-Group Study

**DOI:** 10.3390/ijerph19158936

**Published:** 2022-07-22

**Authors:** Takumi Watanabe, Kyoko Hayashi, Tsuyoshi Takara, Takumi Teratani, Joji Kitayama, Toshio Kawahara

**Affiliations:** 1 Division of Translational Research, Jichi Medical University, 3311-1 Yakushiji, Shimotsuke-shi 329-0498, Tochigi, Japan; teratani@jichi.ac.jp (T.T.); kitayama@jichi.ac.jp (J.K.); 2 College of Life and Health Sciences, Chubu University, 1200 Matsumoto, Kasugai 487-8501, Aichi, Japan; kyhayashi@cronos.ocn.ne.jp (K.H.); toshi@isc.chubu.ac.jp (T.K.); 3 Medical Corporation Seishinkai, Takara Clinic, 2-3-2-9, Higashigotanda, Shinagawa 141-0022, Tokyo, Japan; t-takara@takara-clinic.com

**Keywords:** *Lactiplantibacillus plantarum* SNK12, mental stress, brain–gut axis, lactic acid bacteria

## Abstract

Mouse studies have reported anti-stress effects of *Lactiplantibacillus plantarum* SNK12 (SNK). Specifically, oral SNK administration increased mRNA levels of hippocampal neurotrophic factor and gamma-aminobutyric acid receptor in mice with sub-chronic mild stress-induced social defeat; moreover, it improved depressive behavior. We aimed to evaluate the efficacy of SNK ingestion against stress in healthy adults. We used the Uchida–Kraepelin test for the stress load, with a low-dose (50 mg/day), high-dose (150 mg/day), and placebo groups (dextrin). The primary outcome was the psychological evaluation as measured by the Profile of Mood States 2nd Edition (POMS2) using total mood disturbance (TMD) scores. The secondary outcomes were the score of each POMS2 item, salivary cortisol as a stress marker, and autonomic balance with the low frequency (LF)/ high frequency (HF) ratio. Compared with the placebo group, the SNK ingestion group showed significantly lower TMD scores. Additionally, compared with the placebo group, the high-dose group showed significantly lower scores for Tension-Anxiety and Confusion-Bewilderment, while the low-dose group showed significantly lower Anger-Hostility scores, salivary cortisol levels, and LF/HF scores. Our findings suggest that SNK ingestion could relieve stress (negative feelings, anxiety, tension, embarrassment, confusion, anger, and hostility) resulting from the temporary load caused by work and study.

## 1. Introduction

Mental stress directly increases the risk of psychiatric disorders and exacerbates symptoms; additionally, it indirectly causes gastrointestinal tract inflammation due to stress stimuli transmission through the autonomic nervous system [1,2,3] and increases the risk of developing lifestyle-related diseases, including infectious diseases, obesity, and type II diabetes, due to the weakened immune and endocrine systems [4,5].

The intestinal tract is the largest immune organ covered by mucosal epithelium; moreover, it is responsible for nutrient absorption. Furthermore, the intestinal tract is considered the second brain since the enteric nervous system autonomously controls gastrointestinal motility as well as the transport of water and electrolytes without requiring central control [6,7]. The bidirectional communication system between the brain and intestines is crucially involved in maintaining intestinal homeostasis and brain function [7,8]. The intestinal microbial community influences this communication system through immune, endocrine, and neural pathways [9]. Specifically, gut bacteria significantly impact brain function, which affects mood, cognition, and behavior [10]. Additionally, changes in gut microbiota and microbial stimulation from the intestinal tract are important factors in the “brain–gut axis”, in which the brain and gut are closely linked. Reducing mental stress from the perspective of the intestinal tract through food is an attractive approach, as it could allow reduced medical expenses and extended healthy life expectancy.

Resultingly, active research and development of functional foods aimed at reducing stress has been undertaken. In addition, mental stress is increasing worldwide due to the recent COVID-19 scare, restrictions in daily life to prevent transmission of infections, and the effects of war. Specifically, there has been extensive research on the effects of probiotics, especially lactic acid bacteria and bifidobacteria, which could promote mental health. Administering probiotics can alleviate stress-induced visceral pain and behavior by modulating negative feedback control of the hypothalamic–pituitary–adrenal axis via the hippocampus [11,12]; moreover, it relieves mood disorders by transmitting signals to the brain via the afferent vagus nerve [13]. Additionally, probiotic intake improves human mental health; however, studies have used different experimental methods to evaluate this phenomenon [14,15,16]. Furthermore, these products can be easily consumed by healthy individuals since they are aimed at improving health, rather than treating disease etiology, which involves controlling disease mechanisms and restoring health.

Probiotics are defined as live microorganisms that, when administered in appropriate amounts, provide health benefits to the host [17]. Although lactic acid bacteria and bifidobacteria are considered probiotics, even heat-killed lactic acid bacteria and bifidobacteria exhibit beneficial effects, including from viral infections defense [18], reduction of atopic dermatitis symptoms [19], and suppression of fatty liver syndrome caused by high-fat diet intake [20]. Heat-killed lactic acid bacteria have numerous advantages over live lactic acid bacteria, including improved preservation and portability, as well as less contamination in the production line and less effect on taste, which are crucial for daily consumption of lactic acid bacteria. Heat-killed *Lactobacillus plantarum* SNK12 (SNK) used in this study has been reported to protect against influenza virus infection in mice [18]. Furthermore, in mouse studies, SNK ingestion enhanced mRNA expression of neurotrophic factor (BDNF), neurotrophin-3, and gamma-aminobutyric acid (GABA) receptors under stress-free and chronic social stress conditions, and showed a tendency to improve learning ability and depression-like behavior [21]. Therefore, this study was conducted to investigate whether SNK can be effective in reducing mental stress in humans.

This randomized, placebo-controlled, double-blind, parallel-group study used a stress-loading model. Two groups were determined based on the ingestion of two different doses of SNK (low and high doses of SNK-containing granules; SNK-L and SNK-H, respectively) and were then compared to a placebo group (SNK-free granules as placebos) to clarify the efficacy of SNK. The Uchida–Kleppelin (U–K) test was used for stress loading. The U–K test is a modified version of the Kraepelin-style math test developed by Uchida Yoko [22]. It is also useful for assessing mental stress since it requires focused effort and attention [23,24]; moreover, it is widely used to assess work aptitude as a mental stressor [25,26,27]. This test has been used for many years as a psychological stress test [25]. The test consists of solving a single-digit addition problem for 15 min, changing lines every minute, followed by a break and another 15 min of similar addition [28]. The primary outcome was the Total Mood Disturbance (TMD) score from the Profile of Mood States 2nd Edition (POMS2) [29,30]. The secondary outcomes were assessed using each of the POMS2 items: Tension-Anxiety (TA), Depression-Dejection (DD), Anger-Hostility (AH), Vigor-Activity (VA), Fatigue-Inertia (FI), Confusion-Bewilderment (CB), and Friendliness (F), as well as salivary cortisol levels [31,32] as a biological stress response and the high frequency (HF)/low frequency (LF) ratio [33,34], which is a measure of heart rate variability that reflects sympathetic and parasympathetic nervous system activity. The safety endpoints were evaluated by urinalysis before and after ingestion, peripheral blood analysis, and interview.

## 2. Materials and Methods

### 2.1. Ethics

This clinical study was approved by the Ethical Committee of the Medical Corporation Seishinkai, Takara Clinic, Tokyo, Japan (2105-01660-0051-27-TC, 25 May 2021) and was based on the ethical standards established in the Helsinki Declaration, the ethical guidelines for medical and biological research involving human subjects of Education, Culture, Sports, Science, and Technology; the Ministry of Health, Labor, and Welfare; and the Ministry of Economy, Trade, and Industry of Japan. Written informed consent was obtained from all participants who received appropriate information related to the study before enrollment. This study was registered with the University Hospital Medical Information Network in Japan Clinical Trials Registry (UMIN000044398, 1 June 2021) and was conducted in compliance with the protocol.

### 2.2. Participants

We enrolled healthy Japanese males and females aged ≥ 20 years based on the following inclusion criteria: (1) approval of admission to the study from the supervising physician, (2) considered by the study investigator as normal based on their Beck Depression Questionnaire score, and (3) a high post-stress load TMD score. The exclusion criteria are listed in Table 1.

### 2.3. Test Supplements

We prepared three types of granule-type powders as test supplements. The placebo granule product (Placebo) contained 1.0 g of dextrin per package, while the SNK-containing granule products contained 0.05 g (100 billion bacteria cells) of SNK + 0.95 g of dextrin (SNK-L) per package and 0.15 g (number of bacteria: 300 billion cells) of SNK + 0.85 g of dextrin (SNK-H) per package. There were no discernible differences in appearance, taste, or smell among the three test supplements.

### 2.4. Study Design

This randomized, double-blind, placebo-controlled, parallel-group study was conducted by a contract research organization, ORTHOMEDICO Inc. (Tokyo, Japan), between June 2021 and October 2021 at the Medical Corporation Seishinkai, Takara Clinic (Tokyo, Japan). The number of participants was calculated using d = 0.91, assuming a large difference in TMD scores under stress after 4 weeks of ingestion between SNK ingestion and placebo groups [35]. The number of participants, calculated using a statistical significance level (α) of 5% and a statistical power (1 − β) of 80%, was determined to be 60 (20 in each group). In addition, the number of participants was set at 66 (22 in each group), bearing in mind the possibility of dropouts and noncompliance with the protocol during the study period. Furthermore, in these reports [29,36], stress was also evaluated using POMS2 in 20 participants. Participants were screened for eligibility before the ingestion period based on the inclusion and exclusion criteria through an interview by a supervising physician. The allocation manager randomly assigned participants in a 1:1:1 ratio to the Placebo, SNK-L, and SNK-H groups by stratified randomization with a computer generator, with the following considerations: (i) measured TMD scores after stress loading, (ii) sex, and (iii) age. The allocation manager was independent of other organizations participating in this study and was not involved in determining participant eligibility, data collection, or data analysis. The manufacturer of test supplements printed a mark on the respective bags enclosing the test supplements. The assignment list was kept secret by the allocation manager until the database was unlocked. The participants, investigators, and study personnel remained blinded throughout the study. The participants ingested one packet of placebo or SNK-containing granule product per day with lukewarm water in the morning for 4 weeks. Further, they were instructed to continue their normal lifestyle throughout the study period. Compliance was monitored using interviews and a diary kept by each participant. The U–K test was used for mental stress load as previously described. The test consists of simple addition of single digits, changing the line every minute, with a break in between, for 15 min each in the first and second halves, for a total of 30 min [28], and has been used for many years as a psychological stress load [25].

### 2.5. Measurement

The post-stress load mood state was measured using the POMS2 Japanese version before and after ingestion. Survey items include TMD scores as well as the scores for each questionnaire item (TA, DD, AH, VA, FI, CB, and F). Autonomic measurements were obtained using an autonomic measurement device (VM302; Fatigue Science Laboratory Inc., Osaka, Japan) to measure the LF, HF, and LF/HF. Saliva cortisol levels were measured using the Cortisol (Saliva) EIA Kit, Shizuoka, Japan. Other physical measurements were performed before ingestion; moreover, urinalysis and peripheral blood analysis were performed as safety endpoints.

### 2.6. Primary Outcome

We measured the post-stress load TMD scores at 4 weeks after ingestion. The TMD scores were obtained by subtracting the VA scores from the sum of the TA, CB, AH, DD, and FI scores [29,30].

### 2.7. Secondary Outcomes

The effects of SNK ingestion were evaluated by measuring the amount of post-ingestion changes in TMD scores; TA, DD, AH, VA, FI, CB, F item scores; salivary cortisol levels [31,32] and the high frequency (HF)/low frequency (LF) ratio [33,34].

### 2.8. Safety Endpoints

Urinalysis, peripheral blood analysis, and a medical questionnaire survey were performed as safety endpoints. All data were obtained before the U–K test. The percentage of cases with notable post-intervention changes in urinalysis and peripheral blood test results was determined.

### 2.9. Statistical Analysis

Statistical analyses were performed by a statistician in the contract research organization using IBM SPSS Statistical software, version 23 (IBM Japan, Ltd., Tokyo, Japan). Background characteristics were demographically aggregated according to the analyzed participants. Student’s *t*-test was used for between-group comparisons of age, height, body weight, body mass index, body fat ratio, systolic blood pressure, diastolic blood pressure, pulse rate, and BDI-2 total score. The chi-square test was used for between-group comparisons according to sex.

After the study was completed, the investigator held a conference for reviewing all cases to determine whether they violated the selection criteria, exclusion criteria, or consent collection, as well as to determine how to handle cases of protocol deviation, discontinuation, etc. Based on the consensus reached in this meeting, the principal investigator and statistician decided on how to handle the cases as well as acceptance or rejection of data.

The full analysis set (FAS) comprised of all participants with the exclusion of those that (1) did not receive the assigned intervention, (2) did not meet the conditions of the target population, (3) did not receive any post-allocation intervention, (4) and lacking post-allocation data. The per-protocol analysis set (PPS) consisted of participants in the FAS with the exclusion of those (1) with <80% intake of test supplements, (2) with behavior significantly undermining the credibility of the test results, (3) who met the exclusion criteria after study enrollment, (4) with known non-compliance during the study period, (5) who ingested food or drugs that could significantly affect the study results, (6) with significant changes in lifestyle activities during the study period, and (7) with other obvious reasons for exclusion. The safety analysis population (SAF) comprised participants who met all of the following criteria: (1) received the assigned interventions, (2) received at least one intervention after allocation, and (3) underwent measurement of at least one safety endpoint after allocation.

The primary and secondary outcomes were determined using the PPS. Data collected at the screening point (before the intake test) were applied as baseline values. Regarding the primary and secondary outcomes (except for each POMS2 item), group comparisons at baseline were performed using analysis of variance (ANOVA) with groups as the factor. Additionally, post-intake group comparisons were performed using a two-way repeated-measures analysis of covariance (ANCOVA) with the pre-intake value as the covariate and groups as the factor. Between-group comparisons of each POMS2 item were compared using Mann–Whitney’s U-test.

The SAF was used for analysis of the safety endpoints. Between-group comparisons of the rate of cases with notable post-intervention changes in urinalysis and peripheral blood test results at each time point were performed using a chi-square test. All statistical analyses were two-tailed, with a significance level of 5%.

## 3. Results

### 3.1. Participant Characteristics

**Figure 1:**
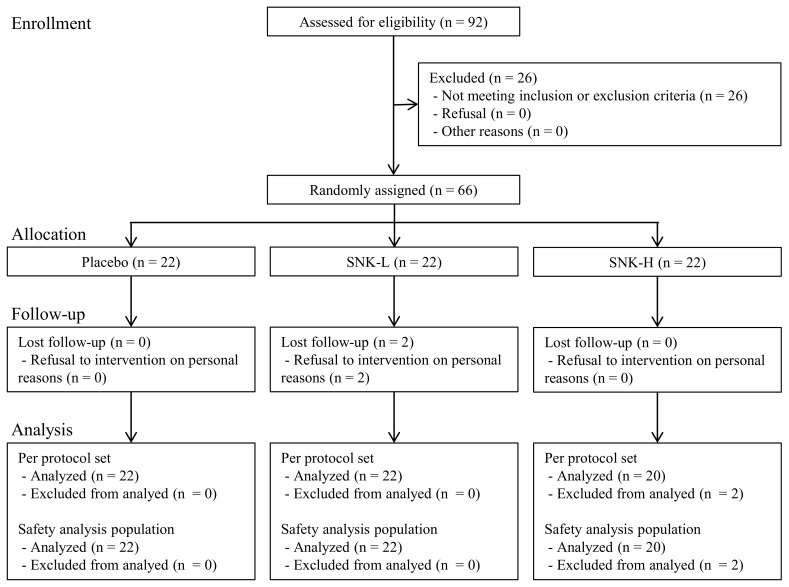
Flow diagram showing participant recruitment and data analysis.

Figure 1 shows the study flow chart of participant recruitment and data analysis. We excluded 26 of 92 screened participants; accordingly, 66 participants (17 men, 49 women) were considered eligible and randomly assigned to the Placebo or SNK-L/H group (n = 22 per group). Subsequently, two participants in the SNK-H group refused to come for personal reasons and were excluded from the PPS and SAF. Accordingly, there were 64 participants in the PPS and SAF (22 in the Placebo group, 22 in the SNK-L group, and 20 in the SNK-H group). Table 2 shows the background characteristics of the participants and pairwise between-group comparisons. There were no medically relevant changes in urinalysis or peripheral blood test values pre- and post-intervention, so there were no problems in conducting the study (Tables S2 and S3).

### 3.2. Primary Outcome

Table 3 shows the mean post-intervention TMD scores, the between-group difference, and standard error (SE) of the Estimated Marginal Means (EMM), and its 95% confidence interval (95% CI^−^, 95% CI^+^). The post-intervention TMD scores in the SNK-L (*p* = 0.047) and SNK-H (*p* = 0.034) groups were significantly lower than those in the Placebo group.

### 3.3. Secondary Outcomes

The post-intervention variations in the TMD scores after stress loading are shown in Table 4. Both the SNK-L (*p* = 0.047) and SNK-H (*p* = 0.034) groups showed significantly lower post-intervention variations in the TMD scores than the Placebo group. Table 5 shows the mean, SD, and amount of variation for each questionnaire item (TA, DD, AH, VA, FI, CB, F) in the POMS2 Japanese version. Compared with the Placebo group, the SNK-H group showed significantly lower TA (*p* = 0.022) and CB (*p* = 0.048) scores while the SNK-L group showed significantly lower AH (*p* = 0.001) scores. Table S1 shows the median and quartile range (Q1: first quartile, Q3: third quartile) of each POMS2 item with significant between-group differences. Compared with the placebo group, the SNK-H group showed significantly lower scores for questions 2 (strain every nerve (*p* = 0.018)) and 42 (sluggish (*p* = 0.026)), while the SNK-L group showed significantly lower scores for questions 2 (strain every nerve (*p* = 0.045)), 3 (get angry (*p* = 0.026)), 14 (grumpy (*p* = 0.042)), and 52 (quick to anger (*p* = 0.031)). Table 6 shows the post-stress loading salivary cortisol levels after 4 weeks of ingestion. The post-intervention salivary cortisol levels were significantly lower in the SNK-L group (*p* = 0.021) than in the Placebo group. Table 7 shows the post-intervention variations in the autonomic measurements (LF, HF, LF/HF). Compared with the Placebo group, the SNK-L group showed significantly lower post-ingestion LF/HF values (*p* = 0.012).

### 3.4. Safety Endpoints

Tables S2 and S3 show the post-intervention changes in urinalysis and peripheral blood test measurements. There were significant differences in lactate dehydrogenase (LDH) levels between the SNK-L and Placebo groups (*p* = 0.043); however, this was confirmed as not a medically problematic change.

## 4. Discussion

In our study, the post-intervention TMD scores and variations were significantly lower in the SNK-L and SNK-H groups compared with the placebo group. This suggested that SNK ingestion was associated with improved mood in response to a mental stress load compared with placebo ingestion. TMD scores were calculated based on a standard sample normalized to have a mean score and SD of 50 and 10, respectively, with lower values indicating better overall mood status [37]. The baseline TMD scores in all groups were above the standard sample mean of 50 points, indicating a negative overall mood. Contrastingly, the post-intervention values in the placebo group remained above the standard sample mean, while those in the SNK-L and SNK-H groups were below the standard sample mean (Table 3), which indicated clinically significant improvement in mood status.

Compared with the placebo group, the SNK-H group showed significantly lower post-intervention scores for TA and CB, while the SNK-L group showed significantly lower AH scores (Table 5). Anxiety, confusion, and anger manifest as psychological stress reactions [31,37]. Since the TMD score is obtained by subtracting the VA score from the sum of the TA, CB, and AH scores as well as the DD and FI scores, which were significantly different as aforementioned, SNK ingestion could have improved overall mood by improving the stress response and suppressing negative emotions, including anxiety, confusion, and anger.

We measured levels of salivary cortisol, a stress hormone, as an objective stress indicator. Compared with the placebo group, the SNK-H and SNK-L groups showed lower salivary cortisol levels, with the SNK-L group showing a significant difference. Mental stress reduction is correlated with decreased cortisol levels [38,39]; moreover, the post-ingestion reduction in salivary cortisol levels is consistent with the post-intervention TMD scores. Probiotic and prebiotic intake has been reported to decrease cortisol levels [40,41,42]; however, few studies have reported significant differences after intake of heat-killed lactic acid bacteria; accordingly, these results could be important. Moreover, since NK cell activity is related to cortisol [40,43], the mechanism of action could be mediated by the immune system. There is a need to elucidate the mechanism of action underlying the anti-stress effects of SNK on the immune system.

Additionally, we obtained objective autonomic measurements. There was a post-ingestion decrease in LF/HF, which was significantly lower in the SNK-L group (1.60 ± 1.46) than in the placebo group (3.66 ± 3.65). The LF/HF value of normal healthy participants was 1.45 [44]; however, the value in the placebo group was 3.66 ± 3.65, which was remarkably high. This suggests that the participants’ sympathetic nervous system was significantly stressed by the load. Contrastingly, the LF/HF value of the SNK-L group was 1.60 ± 1.46, which is close to the average value of healthy participants, and was significantly lower than that of the placebo group (*p* = 0.0119); this indicated relief of load-induced stress. Additionally, the LF/HF value of the SNK-H group (2.36 ± 3.03) was not as high as that of the SNK-L group; however, it tended to be lower than that in the placebo group (*p* = 0.0953). Although SNK could have directly affected the autonomic nervous system, it could have exerted some effect on the brain by influencing the intestinal tract. This suggests that the LF/HF value is consistent with the TMD score.

Fatigue is a widespread and serious problem in the current stressful society given the increasing complexity of social structures and the speed of daily life. Stress is a physiological and psychological strain resulting from physical, chemical, biological, social, and psychological factors. Stress affects the body, mind, and behavior and manifests itself as stress reactions. In Japan, the Ministry of Health, Labor, and Welfare’s “2019 Comprehensive survey of living conditions [45]” and “2020 Occupational health surveillance [46]” reported that 47.9% of individuals aged ≥ 12 years have worries or stress in their daily lives; further, 54.2% of workers reported that several factors in their work or professional life were stressful. This indicates that many workers feel stressed. Stress not only contributes to the development of colitis, lifestyle-related diseases [2,5,47], and sleep disorders, but also induces negative emotions such as irritability, anger, anxiety, and confusion, which manifest as stress responses to cause depression and other mood disorders [2]. Accordingly, there is a need to appropriately deal with stress. Recently, focusing on the brain–gut axis, studies have reported stress response improvement through ingestion of probiotics, including lactic acid bacteria and bifidobacteria [14,15,16,41]. Since BDNF, associated with the onset and pathophysiology of depression and mood disorders, is reduced by stress, stress-induced BDNF reduction may contribute to the onset of depression and other mood disorders [48,49,50]. As aforementioned, *Lactiplantibacillus plantarum* SNK12 ingestion enhances hippocampal BDNF expression and alleviates stress-dependent weight loss [21]. The mechanism of action through which probiotics promote brain BDNF expression remains unclear [51]; however, some probiotics may directly promote BDNF expression in intestinal cells [51,52]. Additionally, butyric acid produced by the intestinal bacterium Clostridium could exert antidepressant effects by increasing BDNF levels in the hippocampus [53,54] and frontal lobe, which suggests that the effects are mediated by changes in the gut microbiota. Consequently, although the mechanism of the stress-relieving effect of SNK is unknown, it has been established that SNK is immune responsive [18]. Therefore, it is suggested that SNK ingestion stimulates intestinal immunity, which changes the gut microbiota, affects the production of short-chain fatty acids, and increases the expression of BDNF, which may improve various negative emotions and overall mood after a temporary mental stress load. Future studies should examine BDNF expression in the intestinal tract to elucidate the mechanisms underlying SNK ingestion. In addition, since BDNF is closely associated with inflammation [55,56], there is a possibility of immune-mediated effects; accordingly, we are considering a comprehensive analysis focusing on intestinal immunity. GABA is related to stress alleviation and has been shown to exert relaxation effects by activating parasympathetic nerves and overall autonomic nervous system in healthy young adult males [57]. Future studies should analyze the dynamics of both GABA and BDNF [21].

Although limited to female participants, the estrogenic effects of isoflavones such as genistein may also have an effect. Genistein is known to reduce various risks, especially in postmenopausal females [58,59,60]. It is assumed that such risk reduction and improvement in quality of life may also affect mental stress. No detailed dietary data were available for this study, but a dietary questionnaire survey was conducted and 28.8% of the participants responded that they consumed soy products daily. Considering this, we postulate that the effect of isoflavones is quite conceivable. It is thought that SNK ingestion may have changed the gut microbiota, resulting in an effect on the ability to convert isoflavone glycosides to aglycons. Therefore, we have planned future studies, intended to analyze the changes in the gut microbiota caused by SNK ingestion, and to further analyze the bacteria involved in the conversion to aglycons.

In addition, we would also like to clarify whether the action is direct (through immune response) or indirect (through intestinal bacteria) by combining with materials related to immunity and inflammation such as polyphenols and non-digestible substances such as oligosaccharides that affect short-chain fatty acids to verify their synergistic effects. If synergistic effects are observed, we postulate that reducing the dosage and making the product available at a low cost would help contribute to reducing the stress levels of the present high-stress society.

Our study has some limitations. No information on the menstrual cycle, which could affect salivary cortisol concentrations, was available for female participants. We also did not obtain information on menopause, although the transition from pre-menopause to post-menopause can have an impact on the body and mind due to hormonal imbalance.

Finally, in the safety analysis, no adverse effects were observed during the study period. Only the post-intervention LDH levels showed significant between-group differences on urinalysis and peripheral blood tests. Specifically, four and zero participants in the SNK-H and placebo groups, respectively, showed fluctuations in LDH levels higher than the reference value (Table S3). However, LDH is increased in various diseases, including cardiac and liver diseases, and is not highly specific; therefore, clinical symptoms and other laboratory findings must be considered. Nonetheless, all observed changes after SNK ingestion were considered as not medically problematic. This suggests that SNK ingestion under the conditions of this study was safe.

## 5. Conclusions

Our findings suggest that ingestion of 50 mg (100 billion cells) or 150 mg (300 billion cells) SNK could relieve stress (negative feelings, anxiety, tension, embarrassment, confusion, anger, hostility) resulting from temporary strain caused by work or study. As for the optimal dose, SNK ingestion at 50 mg/day showed significant differences in TMD scores and salivary cortisol compared to the placebo group, indicating that a dose of 50 mg/day or more is necessary. In the next study, we would like to use a crossover design with lower doses than those used in this study to better define the optimal dose and efficacy. Therefore, to some extent, SNK ingestion can help improve people’s quality of life. We hope that SNK can make a small contribution in alleviating the behavioral restrictions and psychological anxiety caused by COVID-19 in particular. Future studies are warranted to verify the mechanism of action underlying SNK, as well as to clarify the component (nucleic acid, protein, cell wall, etc.) of the lactic acid bacteria that acts as the active ingredient. In the future, we hope to normalize the use of live lactic acid bacteria and bifidobacteria and sterilized lactic acid bacteria and bifidobacteria by clarifying the active ingredients, as well as to make lactic acid bacteria and bifidobacteria more accessible to the public.

## Figures and Tables

**Table 1:** Exclusion criteria.

1	Participants who were under treatment or have a history of malignancy, heart failure, myocardial infarction, psychiatric disorders, immunocompromised participants, short bowel syndrome, ulcerative colitis, valvular heart disease, or bowel obstruction
2	Participants with implanted pacemakers or implantable defibrillators
3	Participants who were under treatment or had experienced chronic diseases (e.g., arrhythmia, hepatopathy, nephropathy, cerebrovascular disorder, rheumatism, diabetes, dyslipidemia, hypertension)
4	Participants who consume foods with possible functional properties on a regular basis
5	Participants who regularly use medicines (including herbal medicines) or supplements
6	Participants with allergies (to medicines and food related to the tested food)
7	Participants who are pregnant, lactating, or intend to become pregnant during the study period
8	Participants who have participated or will participate in other clinical trials during the 28 days prior to the date of consent form acquisition
9	Participants with irregular sleeping hours and sleeping habits due to night shifts, etc.
10	Participants who smoke
11	Participants with infant children
12	Participants who were judged as unsuitable for other reasons by the supervising physician

**Table 2:** Characteristics of the study participants.

	Placebo	SNK-L	SNK-H	*p*-Values (vs. Placebo)	
				SNK-L	SNK-H
Number of Participants (male/female)	22 (5/17)	22 (6/16)	20 (6/14)	1.0000	0.7298
Age (years)	36.0 ± 12.7	38.5 ± 12.0	38.1 ± 10.3	0.4830	0.5650
Height (cm)	161.9 ± 7.1	162.8 ± 9.2	162.1 ± 8.7	0.7400	0.9490
Body weight (kg)	56.3 ± 7.5	59.4 ± 13.2	56.4 ± 11.5	0.3465	0.9802
BMI (kg/m^2^)	21.5 ± 2.6	22.3 ± 4.1	21.3 ± 2.8	0.3887	0.8502
Body fat ratio (%)	26.3 ± 8.2	27.0 ± 9.9	25.6 ± 7.7	0.7993	0.7772
Systolic blood pressure (mmHg)	111.5 ± 10.8	115.2 ± 13.0	114.1 ± 14.1	0.3379	0.5156
Diastolic blood pressure (mmHg)	73.0 ± 8.3	75.0 ± 9.4	75.3 ± 8.9	0.4479	0.4052
Pulse rate (bpm)	72.6 ± 10.0	70.1 ± 10.1	73.5 ± 10.3	0.4161	0.7835
BDI-2 total score (point)	13.8 ± 9.5	15.4 ± 7.6	11.8 ± 9.5	0.5664	0.4653

Between-group comparisons were performed using the χ^2^ test for the number of participants (male/female). Other data are expressed as mean ± standard deviation (SD), with between-group comparisons using ANCOVA.

**Table 3:** Post-stress loading TMD scores after the 4-week intervention.

	Mean	SD	Between-Group Comparison (vs. Placebo)				
			EMM Group Differences	SE	95% CI^−^	95% CI^+^	*p*-Values
Placebo	52.32	11.89					
SNK-L	48.77	7.34	−3.72	1.84	−7.40	−0.05	0.0472 *
SNK-H	48.55	9.51	−4.09	1.88	−7.86	−0.32	0.0338 *

Data are expressed as mean ± SD, EMM group differences, SE of group differences (Placebo vs. SNK-L; Placebo vs. SNK-H), and 95% confidence intervals by ANCOVA with Placebo value as a factor (*, *p* < 0.05).

**Table 4:** TMD scores after stress loading (pre- and post-intervention).

	Placebo		SNK-L			SNK-H		
	Mean	SD	Mean	SD	*p*-Value (vs. Placebo)	Mean	SD	*p*-Value (vs. Placebo)
Pre-intervention	52.09	10.88	52.32	7.69	0.9387	52.50	10.46	0.8925
Post-intervention	52.32	11.89	48.77	7.34	0.0472 *	48.55	9.51	0.0338 *
Variation	0.23	6.28	−3.55	6.72	0.0472 *	−3.95	6.15	0.0338 *

Data are expressed as mean ± SD. Between-group comparisons of pre-intervention values were performed using ANOVA. Between-group comparisons of post-intervention values and variations were performed using ANCOVA, with the pre-intervention value as a factor (*, *p* < 0.05; Placebo vs. SNK-L; Placebo vs. SNK-H).

**Table 5:** Profile of mood states after stress loading.

		Placebo		SNK-L			SNK-H		
		Mean	SD	Mean	SD	*p*-Value (vs. Placebo)	Mean	SD	*p*-Value (vs. Placebo)
TA	Pre-intervention	54.09	11.28	51.09	5.89	0.3095	53.90	11.13	0.9495
	Post-intervention	54.18	12.26	49.27	7.65	0.2624	48.25	11.30	0.0218 *
	Variation	0.09	7.58	−1.82	8.81	0.2624	−5.65	8.60	0.0218 *
DD	Pre-intervention	50.45	9.06	53.18	10.00	0.3560	51.60	10.12	0.7044
	Post-intervention	50.23	9.82	50.91	8.33	0.4583	49.10	9.17	0.2763
	Variation	−0.23	7.21	−2.27	6.56	0.4583	−2.50	4.74	0.2763
AH	Pre-intervention	48.00	10.55	48.27	9.23	0.9271	47.95	9.70	0.9869
	Post-intervention	49.50	11.51	43.59	6.22	0.0013 **	46.35	8.77	0.0975
	Variation	1.50	6.01	−4.68	8.28	0.0013 **	−1.60	5.03	0.0975
VA	Pre-intervention	50.59	14.64	46.64	6.62	0.2185	47.15	8.47	0.2953
	Post-intervention	50.05	10.49	50.36	7.87	0.2218	49.45	10.87	0.5045
	Variation	−0.55	7.84	3.73	4.75	0.2218	2.30	11.37	0.5045
FI	Pre-intervention	57.59	10.94	51.68	10.96	0.0713	51.90	10.06	0.0896
	Post-intervention	55.09	11.05	49.36	10.16	0.4119	49.45	9.12	0.4093
	Variation	−2.50	9.38	−2.32	8.01	0.4119	−2.45	7.84	0.4093
CB	Pre-intervention	53.45	12.27	53.86	9.77	0.9060	55.05	12.21	0.6535
	Post-intervention	53.64	14.57	51.45	8.58	0.2755	50.15	9.64	0.0484 *
	Variation	0.18	8.65	−2.41	7.27	0.2755	−4.90	8.01	0.0484 *
F	Pre-intervention	47.86	15.51	46.45	8.84	0.7096	47.90	12.20	0.9925
	Post-intervention	50.05	12.92	48.73	8.79	0.8607	48.10	11.67	0.4352
	Variation	2.18	7.83	2.27	7.40	0.8607	0.20	12.18	0.4352

Data are expressed as means ± SD. Between-group comparisons of pre-intervention values were performed using ANOVA. Between-group comparisons of post-intervention values and variations were performed using ANCOVA, with the pre-intervention value as a factor. (*, *p* < 0.05; **, *p* < 0.01; Placebo vs. SNK-L; Placebo vs. SNK-H).

**Table 6:** Salivary cortisol levels after stress loading.

	Placebo		SNK-L			SNK-H		
	Mean	SD	Mean	SD	*p*-Value (vs. Placebo)	Mean	SD	*p*-Value (vs. Placebo)
Pre-intervention	0.36	0.14	0.37	0.14	0.8730	0.36	0.12	0.9573
Post-intervention	0.41	0.24	0.29	0.11	0.0205 *	0.31	0.14	0.0586
Variation	0.05	0.24	−0.07	0.16	0.0205 *	−0.05	0.16	0.0586

Data are expressed as means ± SD. Between-group comparisons of pre-intervention values were performed using ANOVA. Between-group comparisons of post-intervention values and variations were performed using ANCOVA, with the pre-intervention value as a factor. (*, *p* < 0.05; Placebo vs. SNK-L; Placebo vs. SNK-H).

**Table 7:** Autonomic nerve balance (LF/HF) after stress loading.

		Placebo		SNK-L			SNK-H		
		Mean	SD	Mean	SD	*p*-Value (vs. Placebo)	Mean	SD	*p*-Value (vs. Placebo)
LF	Pre-intervention	706.23	649.73	535.09	554.61	0.4700	775.05	1072.52	0.7764
	Post-intervention	642.77	518.27	493.14	573.36	0.6136	899.45	1071.32	0.2929
	Variation	−63.45	717.31	−41.95	747.32	0.6136	124.40	1323.53	0.2929
HF	Pre-intervention	484.45	394.20	366.91	355.20	0.6537	732.10	1445.44	0.3576
	Post-intervention	418.73	506.31	580.41	884.77	0.2616	692.70	624.95	0.3547
	Variation	−65.73	432.40	213.50	658.54	0.2616	−39.40	1166.75	0.3547
LF/HF	Pre-intervention	3.00	2.84	2.83	2.71	0.8554	3.06	3.58	0.9498
	Post-intervention	3.66	3.65	1.60	1.46	0.0119 *	2.36	3.03	0.0953
	Variation	0.67	3.89	−1.23	2.46	0.0119 *	−0.70	2.56	0.0953

Data are expressed as means ± SD. Between-group comparisons of pre-intervention values were performed using ANOVA. Between-group comparisons of post-intervention values and variations were performed using ANCOVA, with the pre-intervention value as a factor (*, *p* < 0.05; Placebo vs. SNK-L; Placebo vs. SNK-H).

## Data Availability

Data are provided within the article.

## Supplementary Materials

The following supporting information can be downloaded at: https://www.mdpi.com/article/10.3390/ijerph19158936/s1, Table S1: Post-stress loading POMS2 Questionnaire Items after the 4-week intervention; Table S2: Urinalysis; Table S3: Peripheral blood analysis.
