# Supplementary material for: Effect of Oral Administration of Lactiplantibacillus plantarum SNK12 on Temporary Stress in Adults: A Randomized, Placebo-Controlled, Double-Blind, Parallel-Group Study
Source: Int J Environ Res Public Health. 2022 Jul 22;19(15):8936. doi: 10.3390/ijerph19158936 (PMC9332698; doi:10.3390/ijerph19158936)
Supplement: Supplementary file 1 [file ijerph-19-08936-s001.zip › ijerph-1778041-supplementary.pdf]

Table S1. Post-stress loading POMS Questionnaire Items after the 4-week intervention.

Question No.		Median	Q1	Q3	Between-group comparison Rank sum test		
					Placebo	SNK-L/H	*p* Value
2	Placebo	2.0	1.0	3.0			
	SNK-L	1.0	1.0	2.0	574.0	416.0	0.0451*
	SNK-H	1.0	1.0	2.0	560.5	342.5	0.0179*
3	Placebo	1.0	0.0	2.0			
	SNK-L	0.5	0.0	1.0	584.5	405.5	0.0262*
	SNK-H	1.0	0.0	2.0	512.0	391.0	0.3160
14	Placebo	1.0	0.0	2.0			
	SNK-L	0.0	0.0	1.0	577.5	412.5	0.0416*
	SNK-H	1.0	0.0	2.0	510.0	393.0	0.3385
42	Placebo	3.0	1.0	3.0			
	SNK-L	2.0	1.0	2.0	572.0	418.0	0.0651
	SNK-H	2.0	0.8	2.0	558.5	344.5	0.0256*
45	Placebo	1.0	0.0	2.0			
	SNK-L	0.0	0.0	1.0	578.0	412.0	0.0305*
	SNK-H	0.0	0.0	1.0	524.0	379.0	0.1651

Data are expressed as median (Median), first quartile (Q1), and third quartile (Q3). Question No.2; strain every nerve, Question No.3; get angry, Question No.14; grumpy, Question No.42; sluggish, Question No.52; quick to anger. Between-group comparisons were performed with Mann-Whitney's U test, and rank sums for each group were expressed. (*, $p < 0.05$).

Table S2. Urinalysis.

Parameters	Reference range	Placebo				SNK-L				SNK-H			
		Pre-intervention period		Post-intervention period		Pre-intervention period		Post-intervention period		Pre-intervention period		Post-intervention period	
		In	Out	In	Out	In	Out	In	Out	In	Out	In	Out
Protein	-	22	0	22	0	22	0	22	0	20	0	20	0
Glucose	-	22	0	22	0	22	0	22	0	20	0	20	0

Urobilinogen	±	22	0	21	1	22	0	22	0	20	0	20	0
Bilirubin	-	22	0	22	0	22	0	22	0	20	0	20	0
pH	5.0 - 7.5	22	0	22	0	22	0	21	1	20	0	20	0
Occult Blood	-	22	0	22	0	22	0	22	0	20	0	20	0
Ketone body	-	22	0	22	0	22	0	22	0	20	0	19	1

Data are expressed as the number of subjects whose parameters were inside or outside the reference ranges. Between-group comparisons were performed using the χ2 test. Within group comparisons between values recorded before and after the ingestion period were performed by McNemar test. In, inside reference range; Out, outside reference range.

Table S3. Peripheral blood analysis.

Parameters	Reference range	Placebo				SNK-L				SNK-H			
		Pre-intervention period		Post-intervention period		Pre-intervention period		Post-intervention period		Pre-intervention period		Post-intervention period	
		In	Out	In	Out	In	Out	In	Out	In	Out	In	Out
WBC (/μL)	3300-9000	22	0	20	2	22	0	22	0	20	0	20	0
RBC (x10^4/μL)	Male: 430-570 Femail: 380-500	22	0	22	0	22	0	20	2	20	0	19	1
Hb (g/dL)	Male: 13.5-17.5 Femail: 11.5-15.0	22	0	21	1	22	0	21	1	20	0	20	0
Ht (%)	Male: 39.7-52.4 Femail: 34.8-45.0	22	0	22	0	22	0	21	1	20	0	20	0
Plt (x10^4/μL)	14.0-34.0	22	0	20	2	22	0	21	1	20	0	18	2
MCV (fL)	85-102	22	0	22	0	22	0	22	0	20	0	20	0
MCH (pg)	28.0-34.0	22	0	21	1	22	0	21	1	20	0	20	0
MCHC (%)	30.2-35.1	22	0	21	1	22	0	22	0	20	0	19	1

Neutrophil rate (%)	40.0-75.0	22	0	20	2	22	0	22	0	20	0	20	0
Lymphocyte rate (%)	18.0-49.0	22	0	20	2	22	0	22	0	20	0	20	0
Monocytes (/μL)	-	22	0	21	1	22	0	21	1	20	0	20	0
Eosinophils (/μL)	-	22	0	21	1	22	0	21	1	20	0	20	0
Basophils (/μL)	-	22	0	21	1	22	0	22	0	20	0	20	0
AST (U/L)	10-40	22	0	22	0	22	0	22	0	20	0	19	1
ALT (U/L)	5-45	22	0	22	0	22	0	22	0	20	0	20	0
γ -GTP (U/L)	Male: ≤ 80 Femail: ≤30	22	0	21	1	22	0	21	1	20	0	19	1
ALP (U/L)	100-325	22	0	22	0	22	0	21	1	20	0	19	1
LDH (U/L)	120-240	22	0	22	0	22	0	19	3	20	0	16*	4*
LAP (U/L)	Male: 45-81 Femail: 37-61	22	0	22	0	22	0	22	0	20	0	19	1
Total bilirubin (mg/dL)	0.2-1.2	22	0	22	0	22	0	20	2	20	0	19	1
Direct bilirubin (mg/dL)	0.0-0.2	22	0	22	0	22	0	22	0	20	0	20	0
Indirect bilirubin (mg/dL)	0.2-1.0	22	0	21	1	22	0	21	1	20	0	19	1
Cholinesterase (U/L)	Male: 234-493 Femail: 200-452	22	0	22	0	22	0	22	0	20	0	20	0
Total protein (g/dL)	6.7-8.3	22	0	22	0	22	0	21	1	20	0	20	0
Urea nitrogen (mg/dL)	8.0-20.0	22	0	22	0	22	0	20	2	20	0	19	1
Creatinine	Male: 0.61-1.04	22	0	22	0	22	0	22	0	20	0	19	1

(mg/dL)	Femail: 0.47-0.79												
Uric acid (mg/dL)	Male: 3.8-7.0 Femail: 2.5-7.0	22	0	22	0	22	0	22	0	20	0	19	1
CK (U/L)	Male: 60-270 Femail: 40-150	22	0	21	1	22	0	19	3	20	0	20	0
Na (mEq/L)	137-147	22	0	22	0	22	0	22	0	20	0	20	0
K (mEq/L)	3.5-5.0	22	0	21	1	22	0	20	2	20	0	18	2
Cl (mEq/L)	98-108	22	0	20	2	22	0	21	1	20	0	19	1
Ca (mg/dL)	8.4-10.4	22	0	22	0	22	0	22	0	20	0	20	0
Inorganic phosphorus (mg/dL)	2.5-4.5	22	0	21	1	22	0	21	1	20	0	20	0
Fe (μg/dL)	Male: 50-200 Femail: 40-180	22	0	20	2	22	0	19	3	20	0	20	0
Amylase (U/L)	40-122	22	0	22	0	22	0	20	2	20	0	20	0
Total cholesterol (mg/dL)	120-219	22	0	18	4	22	0	19	3	20	0	18	2
HDL-cholesterol (mg/dL)	Male: 40-85 Femail: 40-95	22	0	21	1	22	0	21	1	20	0	19	1
LDL-cholesterol (mg/dL)	65-139	22	0	18	4	22	0	21	1	20	0	20	0
TG (mg/dL)	30-149	22	0	21	1	22	0	19	3	20	0	19	1
Glucose (mg/dL)	70-109	22	0	22	0	22	0	20	2	20	0	20	0
HbA1c (%)	4.6-6.2	22	0	22	0	22	0	22	0	20	0	20	0
Glycoalbumin (%)	12.3-16.5	22	0	20	2	22	0	22	0	20	0	18	2

Data are expressed as the number of subjects whose parameters were inside or outside the reference ranges. Between-group comparisons were performed using the $\chi2$ test. Within group comparisons between values recorded before and after the ingestion period were performed by McNemar test (*, $p < 0.05$). In, inside reference range; Out, outside reference range.
